# Doravirine/Lamivudine/Tenofovir Disoproxil Fumarate (TDF) Versus Efavirenz/Emtricitabine/TDF in Treatment-naive Adults With Human Immunodeficiency Virus Type 1 Infection: Week 96 Results of the Randomized, Double-blind, Phase 3 DRIVE-AHEAD Noninferiority Trial

**DOI:** 10.1093/cid/ciaa822

**Published:** 2020-12-18

**Authors:** Chloe Orkin, Kathleen E Squires, Jean-Michel Molina, Paul E Sax, Otto Sussmann, Gina Lin, Sushma Kumar, George J Hanna, Carey Hwang, Elizabeth Martin, Hedy Teppler

**Affiliations:** 1 Queen Mary University of London, London, United Kingdom; 2 Thomas Jefferson University, Philadelphia, Pennsylvania, USA; 3 Merck & Co., Inc., Kenilworth, New Jersey, USA; 4 University of Paris Diderot, Hôpital Saint-Louis, Assistance Publique Hôpitaux de Paris, INSERM, Paris, France; 5 Brigham and Women’s Hospital, Harvard Medical School, Boston, Massachusetts, USA; 6 Asistencia Cientifica de Alta Complejidad SAS, Bogota, Colombia

**Keywords:** doravirine, efavirenz, NNRTI, HIV-1, treatment-naive

## Abstract

**Background:**

Doravirine (DOR) is a nonnucleoside reverse-transcriptase inhibitor. In the phase 3 DRIVE-AHEAD trial in treatment-naive adults with human immunodeficiency virus type 1 (HIV-1) infection, DOR demonstrated noninferior efficacy compared with efavirenz (EFV) and superior profiles for neuropsychiatric tolerability and lipids at 48 weeks. We present data through week 96.

**Methods:**

DRIVE-AHEAD is a phase 3, multicenter, double-blind, noninferiority trial in antiretroviral treatment-naive adults with HIV-1 RNA ≥1000 copies/mL. Participants were randomized to a daily fixed-dose tablet of DOR (100 mg), lamivudine (3TC; 300 mg) and tenofovir disoproxil fumarate (TDF; 300 mg) (DOR/3TC/TDF) or EFV (600 mg), emtricitabine (FTC; 200 mg) and TDF (300 mg) (EFV/FTC/TDF). The efficacy end point of interest at week 96 was the proportion of participants with HIV-1 RNA levels <50 copies/mL (Food and Drug Administration Snapshot Approach) with a predefined noninferiority margin of 10% to support week 48 results. Safety end points of interest included prespecified neuropsychiatric adverse events and the mean change in fasting lipids at week 96.

**Results:**

Of 734 participants randomized, 728 received study drugs and were included in analyses. At week 96, HIV-1 RNA <50 copies/mL was achieved by 77.5% of DOR/3TC/TDF vs 73.6% of EFV/FTC/TDF participants, with a treatment difference of 3.8% (95% confidence interval, –2.4% to 10%). Virologic failure rates were low and similar across treatment arms, with no additional resistance to DOR observed between weeks 48 and 96. Prespecified neuropsychiatric adverse events and rash were less frequent in DOR/3TC/TDF than in EFV/FTC/TDF participants through week 96. At week 96, fasting low-density lipoprotein cholesterol and non–high-density lipoprotein cholesterol (HDL-C) levels increased in the EFV/FTC/TDF group but not in the DOR/3TC/TDF group; the mean changes from baseline in total cholesterol/HDL-C ratio were similar.

**Clinical Trials Registration:**

NCT02403674.

## Teaser

Week 96 results support noninferior efficacy of doravirine (DOR)/3TC/ tenofovir disoproxil fumarate (TDF) compared with efavirenz (EFV)/ emtricitabine (FTC)/TDF established at week 48. DOR/3TC/TDF was well tolerated, with fewer neuropsychiatric and rash events than EFV/FTC/TDF.

Doravirine (DOR) is a nonnucleoside reverse-transcriptase inhibitor (NNRTI) for the treatment of human immunodeficiency virus type 1 (HIV-1) [1, 2]. DOR is active in vitro against both wild-type HIV-1 and most common NNRTI-resistant variants (K103N, Y181C, G190A, K103N/Y181C, and E138K) at concentrations achieved with 100-mg once-daily dosing [3]. DOR has a unique in vitro resistance profile among NNRTIs, with lower median inhibitory concentration (IC_50_) values against NNRTI-resistant variants than other NNRTIs [3–5]. DOR can be taken without regard to food, which does not have an important effect on DOR’s pharmacokinetics [6, 7]. In addition, DOR has a low potential for drug-drug interactions, including with acid-reducing agents, statins, and metformin [7]. DOR is currently approved as a single-entity tablet to be administered in combination with other antiretroviral drugs, and a once-daily fixed-dose combination tablet containing DOR (100 mg), lamivudine (3TC; 300 mg), and tenofovir disoproxil fumarate (TDF; 300 mg) (DOR/3TC/TDF) [8].

In the phase 3 DRIVE-FORWARD trial, DOR demonstrated noninferior efficacy and a superior lipid profile compared with darunavir-ritonavir at 48 weeks of combination treatment with 2 nucleoside reverse-transcriptase inhibitors (NRTIs) [9]. In the phase 3 DRIVE-AHEAD trial, DOR/3TC/TDF demonstrated efficacy noninferior to efavirenz (EFV; 600 mg) emtricitabine (FTC; 200 mg), and TDF (300 mg) (EFV/FTC/TDF) at week 48 and was well tolerated, with significantly fewer neuropsychiatric adverse events (AEs) and minimal changes in low-density lipoprotein cholesterol and non–high-density lipoprotein cholesterol (HDL-C) compared with EFV/FTC/TDF [10]. Because durability of virologic response and long-term safety and tolerability are critical factors when evaluating a novel HIV-1 treatment, the double-blind period of the DRIVE-AHEAD trial was planned for a total of 96 weeks; herein we report the results of the DRIVE-AHEAD trial through week 96.

## METHODS

### Trial Design

**Figure 1.**
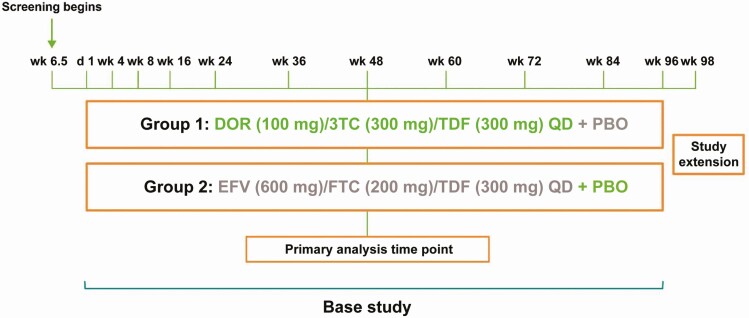
Trial design. Abbreviations: 3TC, lamivudine; DOR, doravirine; EFV, efavirenz; FTC, emtricitabine; PBO, placebo; QD, once daily; TDF, tenofovir disoproxil fumarate.

DRIVE-AHEAD (MK-1439A protocol 021; NCT02403674) is a randomized, active-controlled, double-blind, noninferiority phase 3 trial in antiretroviral treatment-naive adults with HIV-1 RNA levels ≥1000 copies/mL conducted at 126 global study sites in 23 countries. Full methods have been previously reported in detail with the 48-week results [10]. The trial was conducted in accordance with principles of Good Clinical Practice requirements and was approved by the appropriate institutional review boards and regulatory agencies. Participants were randomly assigned (1:1 by interactive voice response system/interactive Web response system) to a single-tablet regimen of DOR/3TC/TDF (plus placebo for EFV/FTC/TDF) or EFV/FTC/TDF (plus placebo for DOR/3TC/TDF), after stratification according to screening HIV-1 RNA (≤100 000 or >100 000 copies/mL) and chronic hepatitis B and/or C coinfection (yes or no). DOR/3TC/TDF or its matching placebo was taken orally once daily without regard to food at approximately the same time each day for 96 weeks, and EFV/FTC/TDF or matching placebo was taken orally once daily on an empty stomach (Figure 1).

### Assessments

Plasma HIV-1 RNA levels were measured at all study visits by the central laboratory using the Abbott RealTime HIV-1 assay (lower limit of quantification, 40 copies/mL). Protocol-defined virologic failure (PDVF) was defined as nonresponse (HIV-1 RNA ≥200 copies/mL at week 24 or 36 or ≥50 copies/mL at week 48) or viral rebound (HIV-1 RNA ≥50 copies/mL after initial response of HIV-1 RNA <50 copies/mL at any time during the trial). Initial PDVF measurement required an additional confirmatory measurement of HIV-1 RNA taken ≥1 week apart. Participants who met these criteria were discontinued from the trial.

Viral resistance testing to the study drugs was performed for participants with PDVF and those who discontinued the trial for any reason if HIV-1 RNA was >400 copies/mL. Postbaseline genotypic viral resistance to DOR was defined as any of the following mutations in the RT gene: A98G, L100I, K101E, V106A, V106I,V106M, V108I, E138K, Y188L, G190A, G190S, H221Y, P225H, F227C, F227L, F227V, M230I, M230L, L234I, P236L, and Y318F. Postbaseline genotypic resistance to EFV, FTC, 3TC, and TDF was assessed by Monogram Biosciences, LabCorp Specialty Testing Group. Phenotypic viral resistance to EFV and NRTIs was defined by Monogram Biosciences based on the difference (fold change) between the IC_50_ values for a participant’s virus and wild-type virus. Because no threshold for defining phenotypic resistance to DOR was clinically defined, a 2.5-fold change in IC_50_ versus wild-type virus was used as a broad assay reproducibility threshold for potential phenotypic resistance to DOR. To evaluate the immunologic effects of DOR, CD4^+^ T-cell counts were assessed at screening, day 1, and weeks 8, 24, 48, 72, and 96, using flow cytometry at a central laboratory.

Safety was monitored by AE reporting, treatment-emergent laboratory abnormalities, and physical examinations. AEs were assessed by the investigator for intensity (mild, moderate, severe) and relationship to study therapy. Laboratory values were graded in severity based on Division of Acquired Immunodeficiency Syndrome criteria [11]. Detailed statistical methods were previously reported in the week 48 publication [10].

## RESULTS

### Participants

**Figure 2.**
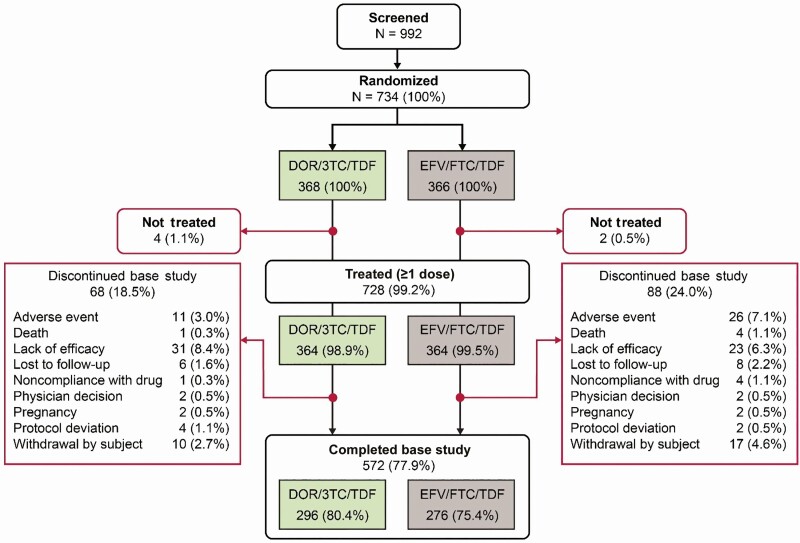
Disposition of participants through week 96. Abbreviations: 3TC, lamivudine; DOR, doravirine; EFV, efavirenz; FTC, emtricitabine; TDF, tenofovir disoproxil fumarate.

A total of 992 participants were screened, of whom 734 were randomized with 368 participants in the DOR/3TC/TDF group and 366 in the EFV/FTC/TDF group. Of the randomized participants, 364 received study medication in each group and were included in both efficacy and safety analyses. Demographics and baseline characteristics were generally similar between the treatment groups (Table 1). A total of 68 participants (18.5%) from the DOR/3TC/TDF group and 88 (24.0%) from the EFV/FTC/TDF discontinued treatment early without completing week 96 of the trial; the most common reasons for discontinuation were lack of efficacy, AEs, and withdrawal of consent (Figure 2). Between weeks 48 and 96, 17 participants discontinued from the DOR/3TC/TDF group, and 27 discontinued from the EFV/FTC/TDF group. In the DOR/3TC/TDF group, discontinuations after week 48 included 13 due to lack of efficacy, 1 due to an AE, 2 due to withdrawal of consent, and 1 due to pregnancy. In the EFV/FTC/TDF group discontinuations after week 48 included 13 due to lack of efficacy, 3 due to an AEs, 6 due to withdrawal of consent, 1 due to pregnancy, 1 lost to follow-up, 2 for nonadherence, and 1 due to death.

**Table 1.** Demographic and Baseline Characteristics

Characteristic	Participants, No. (%)^a^		
	DOR/3TC/TDF	EFV/FTC/TDF	Total
	(n = 364)	(n = 364)	(n = 728)
Age, median (range), y	32.0 (18–70)	30.0 (18–69)	31.0 (18–70)
Male sex	305 (84)	311 (85)	616 (85)
Race or ethnicity			
White	177 (49)	170 (47)	347 (48)
Black or African American	67 (18)	68 (19)	135 (19)
Asian	59 (16)	65 (18)	124 (17)
Other^b^	61 (17)	61 (17)	122 (17)
Hispanic or Latino ethnicity	126 (35)	120 (33)	246 (34)
Region			
Africa	37 (10)	27 (7)	64 (9)
Asia/Pacific	59 (16)	62 (17)	121 (17)
Europe	88 (24)	94 (26)	182 (25)
Latin America	89 (24)	87 (24)	176 (24)
North America	91 (25)	94 (26)	185 (25)
CD4^+^ T-cell count, median (range), cells/μL	414 (19–1399)	388 (19–1452)	397 (19–1452)
CD4^+^ T-cell count			
≤200/μL	44 (12)	46 (13)	90 (12)
>200/μL	320 (88)	318 (87)	638 (88)
Plasma HIV-1 RNA, median (range), log_10_ copies/mL	4.4 (2.4–6.1)	4.5 (2.6–6.4)	4.4 (2.4–6.4)
Plasma HIV-1 RNA			
≤100 000 copies/mL	291 (80)	282 (77)	573 (79)
>100 000 copies/mL	73 (20)	82 (23)	155 (21)
History of AIDS	46 (13)	53 (15)	99 (14)
Hepatitis B and/or C^c^	11 (3)	9 (2)	20 (3)
HIV-1 subtype B	232 (64)	253 (70)	485 (67)

Abbreviations: 3TC, lamivudine; DOR, doravirine; EFV, efavirenz; FTC, emtricitabine; HIV-1, human immunodeficiency virus type 1; TDF, tenofovir disoproxil fumarate.

^a^Data represent no. (%) of participants unless otherwise specified.

^b^“Other” race includes multiracial, American Indian, or Alaska native.

^c^Evidence of hepatitis B surface antigen or hepatitis C virus RNA.

### Efficacy

**Figure 3.**
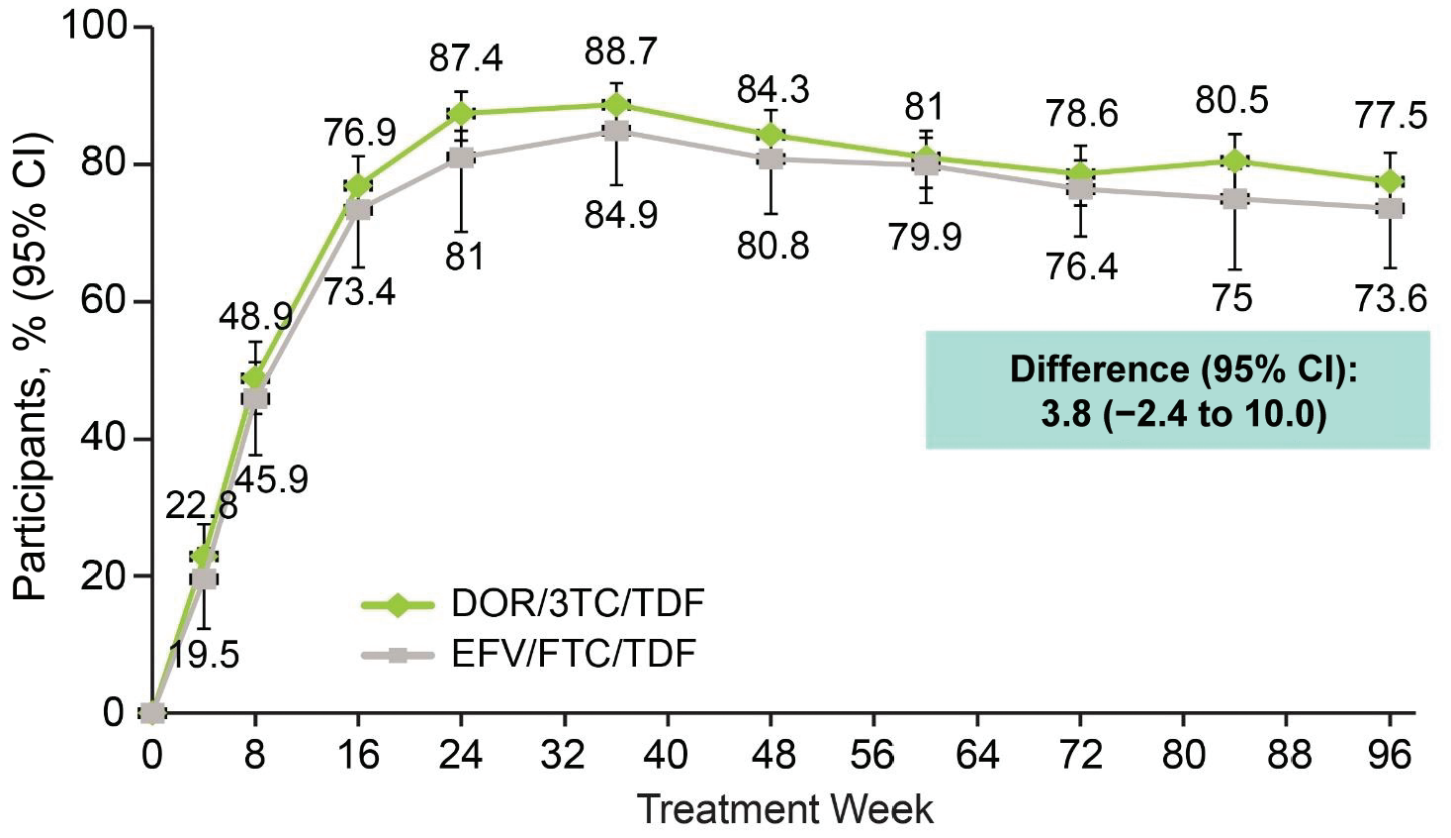
Proportion of participants with human immunodeficiency virus type 1 RNA level <50 copies/mL over time (Food and Drug Administration snapshot approach). Abbreviations: 3TC, lamivudine; CI, confidence interval; DOR, doravirine; EFV, efavirenz; FTC, emtricitabine; TDF, tenofovir disoproxil fumarate.

**Figure 4.**
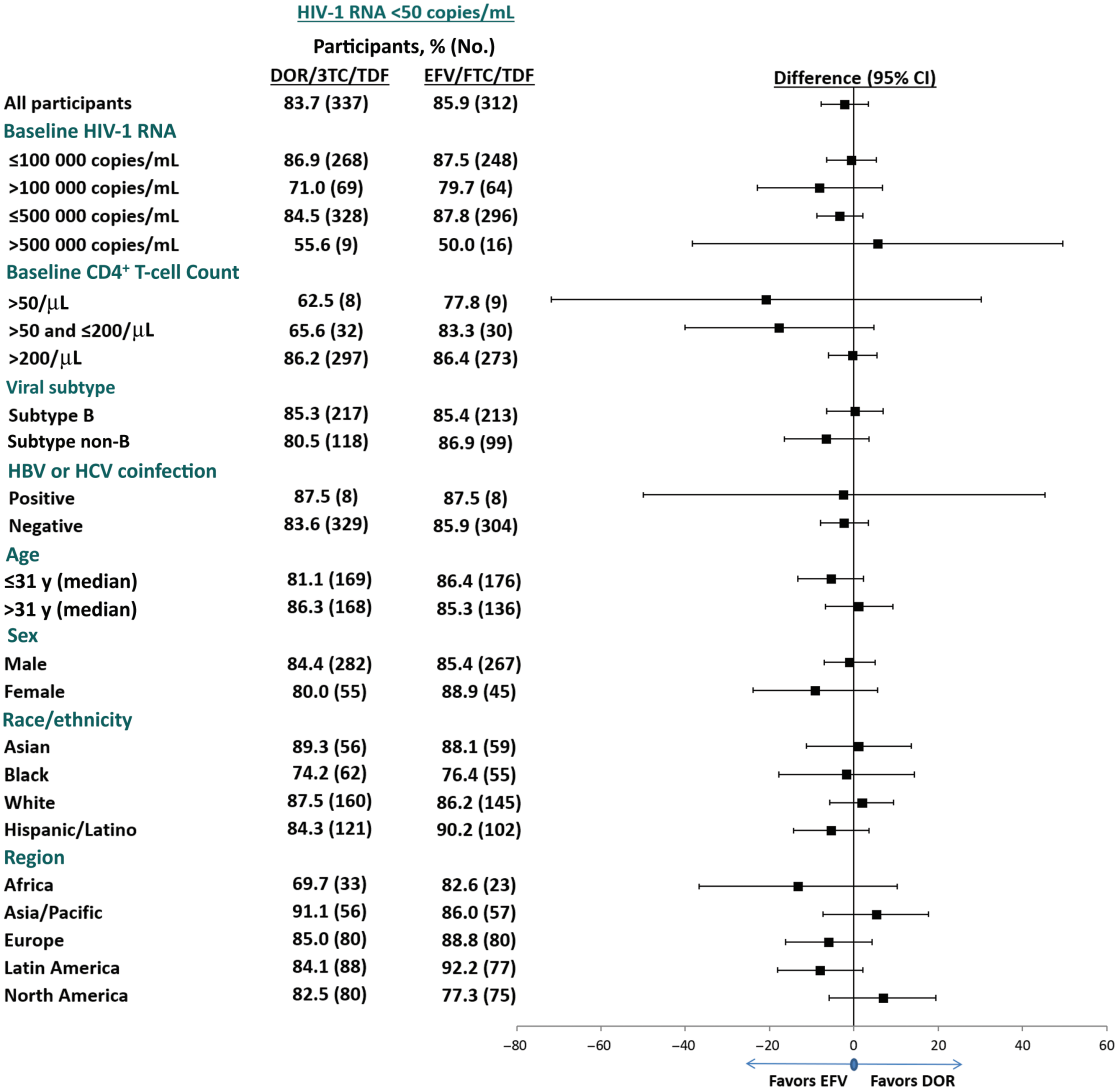
Efficacy by subgroup (observed failure approach) at week 96. Abbreviations: 3TC, lamivudine; CI, confidence interval; DOR, doravirine; EFV, efavirenz; FTC, emtricitabine; HBV, hepatitis B virus, HCV, hepatitis C virus; HIV-1, human immunodeficiency virus type 1; TDF, tenofovir disoproxil fumarate.

At week 96, the proportion of participants with HIV-1 RNA <50 copies/mL was 77.5% (282 of 364) in the DOR/3TC/TDF group and 73.6% (268 of 364) in the EFV/FTC/TDF group (treatment difference, 3.8%; 95% confidence interval (CI), −2.4% to 10.0%) (Food and Drug Administration Snapshot Approach), which supports the noninferiority of DOR/3TC/TDF to EFV/FTC/TDF established at week 48. Virologic response rates were similar between treatment groups at each time point (Figure 3) and across all baseline characteristics and prognostic factors. Similar results were observed for the proportion of participants achieving virologic end point thresholds of HIV-1 RNA <40 copies/mL and <200 copies/mL (Table 2). At week 48, we reported a differential efficacy response for subgroups of participants aged ≤31 or >31 years (the median age); however, this trend was not observed at week 96. Among participants with high baseline HIV-1 RNA levels (>100 000 copies/mL), 49 of 69 (71.0%) in the DOR/3TC/TDF group and 51 of 64 (79.7%) in the EFV/FTC/TDF group achieved HIV-1 RNA levels <50 copies/mL at week 96 (treatment difference, −8.1%; 95% CI, −22.9% to 6.7%) (observed failure approach) (Figure 4).

**Table 2.** Virologic Outcomes at Week 96

Outcome	Participants, No. (%)	
	DOR/3TC/TDF (n = 364)	EFV/FTC/TDF (n = 364)
Primary analysis (FDA snapshot approach)		
HIV-1 RNA <50 copies/mL	282 (77.5)	268 (73.6)
HIV-1 RNA ≥50 copies/mL^a^	55 (15.1)	44 (12.1)
No virologic data in wk 96 window	27 (7.4)	52 (14.3)
Discontinued study due to AE or death^b^	12 (3.3)	30 (8.2)
Discontinued study for other reasons^c^	13 (3.6)	20 (5.5)
On study but missing data in window	2 (0.5)	2 (0.5)
Secondary and exploratory end points		
HIV-1 RNA <50 copies/mL (observed failure)	282/337 (83.7)	268/312 (85.9)
HIV-1 RNA <40 copies/mL (FDA snapshot)	277/364 (76.1)	265/364 (72.8)
HIV-1 RNA <200 copies/mL (FDA snapshot)	292/364 (80.2)	272/364 (74.7)

Abbreviations: 3TC, lamivudine; AE, adverse event; DOR, doravirine; EFV, efavirenz; FDA, Food and Drug Administration; FTC, emtricitabine; HIV-1, human immunodeficiency virus type 1; TDF, tenofovir disoproxil fumarate.

^a^Including participants who changed any component of background therapy before week 96, participants who discontinued a study drug before week 96 for lack or loss of efficacy, and participants with HIV-1 RNA levels ≥50 copies/mL in the week 96 window.

^b^Including participants who discontinued because of AEs or death at any time point from day 1 through the time window if this resulted in no virologic data on treatment during the specified window.

^c^The other reasons included lost to follow-up, noncompliance with study drug, physician decision, pregnancy, protocol deviation, screen failure, and withdrawal by participant.

Among participants with >50 and ≤200 CD4^+^ T-cell count at baseline, 21 of 32 (65.6%) in the DOR/3TC/TDF group and 25 of 30 (83.3%) in the EFV/FTC/TDF group achieved HIV-1 RNA <50 copies/mL at week 96 (treatment difference, −17.7%; 95% CI, −40.0% to 4.6%) (observed failure approach) (Figure 4). Among participants with ≤50 CD4^+^ T-cell count at baseline, 5 of 8 (62.5%) in the DOR/3TC/TDF group and 7 of 9 (77.8%) in the EFV/FTC/TDF group achieved HIV-1 RNA <50 copies/mL at week 96 (treatment difference, −20.8%; 95% CI, −71.8% to 30.1%) (observed failure approach) (Figure 4).The mean change in CD4^+^ T-cell count from baseline to week 96 was similar in the DOR/3TC/TDF and EFV/FTC/TDF groups (238/μL vs 223/μL; treatment difference, 14.7%; 95% CI, −18.7% to 48.2%).

At week 96, the proportions of participants who met the definition of PDVF (rebound and nonresponse) were low and similar in the DOR/3TC/TDF (9.3%) and EFV/FTC/TDF (7.7%) treatment groups, as compared with rates of 5.7% and 3.8%, respectively at week 48. Rates of treatment-emergent drug resistance was low for both DOR/3TC/TDF and EFV/FTC/TDF groups (Table 3). No additional participants in the DOR/3TC/TDF group developed treatment-emergent resistance to DOR after week 48.

**Table 3.** Summary of Treatment Emergent Drug Resistance Through Week 96

	articipants, No. (%)^a^					
Resistance	DOR/3TC/TDF (n = 364)			EFV/FTC/TDF (n = 364)		
	PDVF^b^	Discontinued Without PDVF	Total	PDVF^b^	Discontinued Without PDVF	Total
All participants	34 (9.3)	39 (10.7)	73 (20.0)	28 (7.7)	62 (17.0)	90 (24.7)
Genotype test successful^c^	21 (5.8)	11 (3.0)	32 (8.8)	15 (4.1)	18 (4.9)	33 (9.0)
Phenotype test successful	22 (6.0)	12 (3.3)	34 (9.3)	15 (4.1)	17 (4.7)	32 (8.8)
Resistance type						
Genotypic NNRTI^d^	7 (1.9)	1 (0.3)	8 (2.2)	10 (2.7)	4 (1.1)	14 (3.8)
Phenotypic NNRTI	6 (1.6)	0	6 (1.6)	9 (2.5)	4 (1.1)	13 (3.6)
Genotypic and/or phenotypic NRTI	6 (1.6)	0	6 (1.6)	5 (1.4)	2 (0.5)	7 (1.9)
Specific NNRTI resistance mutations detected^e^	Y188L; Y318Y/F; V106I, H221H/Y, F227C; A98A/G, V106V/I, H221H/Y, F227C; A98A/G, V106V/I, F227F/C; A98A/G, V106A, P225H, Y318Y/F; V106M/T, V108V/I, F227C/R; V106I			K103N; K103N, E138E/G; K103N; K103N; G190E; K103N; K103N, M230L; G190E; K103N, V108V/I, T369T/A/I/V; K103N, V179V/D, N384R; K103N; K103N; K103N; K101K/N, K103N, P225P/H		
Specific NRTI resistance mutations detected^e^	M41L, M184V; M184V; M184V; K65R; K65K/R, M184V; M184M/V			V118I, M184V; M184V; M184V; M184V, K219K/E; K65K/R, M184M/I; V118I		

Abbreviations: 3TC, lamivudine; DOR, doravirine; EFV, efavirenz; FTC, emtricitabine; NNRTI, nonnucleoside reverse-transcriptase inhibitor; NRTI, nucleoside reverse-transcriptase inhibitor; PDVF, protocol-defined virologic failure; TDF, tenofovir disoproxil fumarate.

^a^Data represent no. (%) of participants unless otherwise specified.

^b^PDVF was defined as confirmed human immunodeficiency virus type 1 (HIV-1) RNA ≥50 copies/mL after initial response of HIV-1 RNA <50 copies/mL at any time during the study; confirmed HIV-1 RNA ≥200 copies/mL at week 24 or 36; or confirmed HIV-1 RNA ≥50 copies/mL at week 48.

^c^Genotyping was performed on samples with HIV-1 RNA levels >400 copies/mL

^d^Includes 1 participant who had discontinued due to physician decision at Week 4 not originally reported in the Week 48 publication due to availability of resistance data becoming available after publication.

^e^Mutations detected are listed by participant (with participants’ mutations separated by semicolons).

We do report 1 additional instance of DOR resistance that was not in the previous week 48 publication. Viral drug resistance information for 1 participant in the DOR/3TC/TDF group who had discontinued without PDVF due to a physician decision at week 4 became available after finalization of the week 48 publication. This participant had a genotypic mutation (V106I) known to be associated with NNRTI resistance; phenotype analysis showed that the virus remained susceptible to DOR. In the EFV/FTC/TDF group, 1 additional participant who discontinued due to PDVF developed resistance to EFV after week 48. Two additional participants who discontinued without PDVF were identified with resistance to EFV after week 48; 1 had virus with both genotypic and phenotypic resistance, and the other had virus with an accessory T369V mutation but unsuccessful phenotype testing. In the DOR/3TC/TDF group, of the 8 participants with virus with documented DOR-associated NNRTI resistance mutations, 7 also had virus with documented NRTI resistance. In the EFV/FTC/TDF group, of the 14 participants with virus who had documented EFV-associated NNRTI resistance mutations 6 also had virus with documented NRTI resistance.

### Safety

Overall rates of any AEs, drug-related AEs, and discontinuation due to AEs through week 96 were lower in the DOR/3TC/TDF group than in the EFV/FTC/TDF group, consistent with the results from week 48 (Table 4). Through week 96, a total of 38 participants experienced AEs that resulted in discontinuation from the study (11 [3.0%] in the DOR/3TC/TDF group and 27 [6.6%] in the EFV/FTC/TDF group). This total included 1 additional participant from the DOR/3TC/TDF group (depression) and 3 additional participants who discontinued since the week 48-time point in the EFV/FTC/TDF group (1 each with dizziness/headache/somnolence, sleep disorder, and abnormal dreams). The most common AE that led to discontinuation in the EFV/FTC/TDF group was rash, with 10 participants total; only 1 additional case of rash was reported in the EFV/FTC/TDF group after week 48. No participants in the DOR/3TC/TDF group discontinued due to rash. No AE leading to discontinuation in the DOR/3TC/TDF group occurred in >1 participant.

**Table 4.** Summary of Clinical Adverse Events Through Week 96

AEs	Participants, No. (%)		
	DOR/3TC/TDF (n = 364)	EFV/FTC/TDF (n = 364)	Treatment Difference, % (95% CI)
Any AE	321 (88)	339 (93)	−4.9 (−9.3 to −.7)
Drug-related AE^a^	116 (32)	236 (65)	−33.0 (−39.6 to −26.0)
Serious AE	21 (6)	30 (8)	−2.5 (−6.3 to 1.3)
Drug-related serious AE	1 (<1)	4 (1)	−0.8 (−2.5 to .5)
Deaths^b^	0 (0)	2 (1)	−0.5 (−2.0 to .5)
Discontinued due to AE	11 (3)	27 (7)	−4.4 (−7.9 to −1.2)
Discontinued due to drug-related AE	8 (2)	24 (7)	−4.4 (−7.7 to −1.5)
Neuropsychiatric AE (prespecified)^c^	96 (26)	213 (59)	−32.1 (−38.8 to −25.2)
Most common AEs^d^			
Headache	57 (16)	56 (15)	0.3 (−5.0 to 5.6)
Nasopharyngitis	50 (14)	43 (12)	1.9 (−3.0 to 6.8)
Diarrhea	48 (13)	58 (16)	−2.7 (−7.9 to 2.4)
Upper respiratory tract infection	41 (11)	29 (8)	3.3 (−1.0 to 7.7)
Dizziness	37 (10)	139 (38)	−28.0 (−33.9 to −22.1)
Nausea	31 (9)	42 (12)	−3.0 (−7.5 to 1.4)
Insomnia	25 (7)	38 (10)	−3.6 (−7.8 to .5)
Rash	20 (6)	45 (12)	−6.9 (−11.2 to −2.8)
Abnormal dreams	18 (5)	44 (12)	−7.1 (−11.4 to −3.2)

Abbreviations: 3TC, lamivudine; AE, adverse event; CI, confidence interval; DOR, doravirine; EFV, efavirenz; FTC, emtricitabine; TDF, tenofovir disoproxil fumarate.

^a^AEs determined by the investigator to be related to study therapy.

^b^None of the deaths were considered related to study therapy.

^c^Prespecified AEs included dizziness, sleep disorders and disturbances, altered sensorium, depression and suicide/self-injury, and psychosis and psychotic disorders.

^d^AEs with ≥10% incidence in either treatment group.

The frequency of Division of Acquired Immunodeficiency Syndrome grade 3–4 laboratory abnormalities was low and similar between the treatment groups (Table 5). Laboratory abnormalities that indicate renal dysfunction occurred infrequently in both arms of the study. Fifteen participants (10 in the DOR/3TC/TDF group and 5 in the EFV/FTC/TDF, with a treatment difference of 1.4% [95% CI, −.8% to 3.8%]) had grade 3 creatinine levels, defined as either a >1.8- to <3.5-fold-increase above the upper normal limit or as a 1.5- to <2.0 fold-increase from the patient’s baseline value. All grade 3 creatinine values were <2.0 mg/dL and transient; most of these met grade 3 criteria due to a change from baseline value and remained within the normal range. Two participants (1 in each treatment group) had grade 4 creatinine elevations. One participant in the DOR/3TC/TDF group had a creatinine elevation (to 1.69 mg/dL from 0.83 mg/dL at baseline) on day 595 with no prior elevations. The participant continued in the trial, and creatinine levels returned to below grade 1 by the next study visit. One participant in the EFV/FTC/TDF group had grade 4 creatinine elevation (to 12.63 mg/dL from 0.64 mg/dL at baseline) on day 172 in the setting of moderate diabetic decompensation and with acute renal failure noted the same day; this participant was withdrawn from the trial.

**Table 5.** Most Common Grade 3 or 4 Laboratory Findings Through Week 96^a^

Criterion	Participants Meeting Criterion^b^/Participants With ≥1 Postbaseline Test, No. (%)		Difference, % (95% CI^c^)
	DOR/3TC/TDF	EFV/FTC/TDF	
Fasting LDL-C ≥190 mg/dL (grade 3)	1/332 (0.3)	5/310 (1.6)	−1.3 (−3.5 to .2)
Fasting triglycerides >500 to 1000 mg/dL (grade 3)	2/336 (0.6)	9/319 (2.8)	−2.2 (−4.7 to −.3)
Creatinine >1.8 to <3.5 × ULN, or increase to 1.5 to <2.0 × baseline (in mg/dL; grade 3)	10/363 (2.8)	5/359 (1.4)	1.4 (−.8 to 3.8)
AST 5.0 to <10.0 × ULN (in IU/L; grade 3)	2/363 (0.6)	11/359 (3.1)	−2.5 (−4.9 to −0.7)
ALT 5.0 to <10.0 × ULN (in IU/L; grade 3)	3/363 (0.8)	7/359 (1.9)	−1.1 (−3.2 to 0.7)
Lipase 3.0 to <5.0 × ULN (in IU/L; grade 3)	4/363 (1.1)	7/359 (1.9)	−0.8 (−3.0 to 1.1)
Creatine kinase			
10.0 to < 20.0 × ULN (grade 3)	12/363 (3.3)	12/359 (3.3)	−0.0 (−2.8 to 2.7)
≥20.0 × ULN (grade 4)	4/363 (1.1)	10/359 (2.8)	−1.7 (−4.1 to 0.4)

Abbreviations: 3TC, lamivudine; ALT, alanine aminotransferase; AST, aspartate aminotransferase; CI, confidence interval; DOR, doravirine; EFV, efavirenz; FTC, emtricitabine; LDL-C, low-density lipoprotein cholesterol; TDF, tenofovir disoproxil fumarate; ULN, upper limit of normal range.

^a^The most common findings were defined as those occurring in ≥4 participants in either treatment group.

^b^Participants are counted once per test in the highest grade reported. Only participants with a worsened grade relative to baseline are included.

^c^The 95% CIs were calculated using Miettinen and Nurminen method.

**Figure 5.**
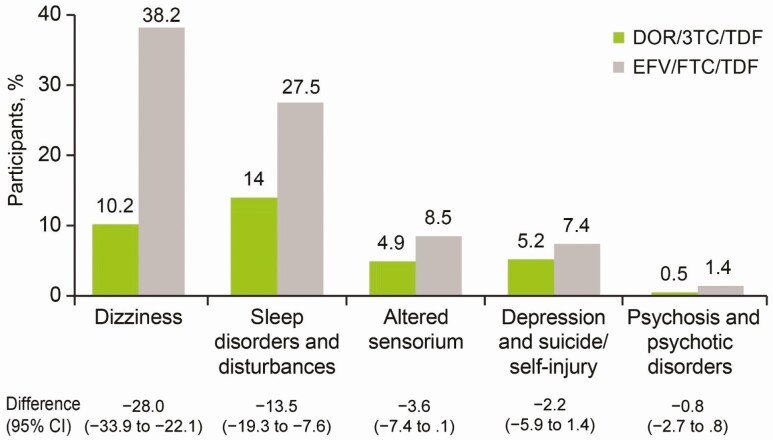
Proportion of participants with neuropsychiatric adverse events in prespecified categories through week 96. Abbreviations: 3TC, lamivudine; CI, confidence interval; DOR, doravirine; EFV, efavirenz; FTC, emtricitabine; TDF, tenofovir disoproxil fumarate. (Between-group difference calculated as follows: DOR – EFV.)

At week 48, the primary safety end points were the proportions of participants with prespecified neuropsychiatric events, which were significantly lower in the DOR/3TC/TDF group than in the EFV/FTC/TDF group. Consistent with results from weeks 0–48, overall neuropsychiatric AEs (weeks 0–96) were less common for DOR/3TC/TDF (26.4%) than for EFV/FTC/TDF (58.5%) (treatment difference, −32.1%; 95% CI, −38.8% to −25.2%). By category, the 95% CI for the treatment difference excluded 0 for 2 of the 5 predefined categories: dizziness and sleep disorders/disturbances (Figure 5). The majority of reported neuropsychiatric AEs occurred before week 48 for both groups, with minimal difference in new-onset neuropsychiatric AEs between groups after week 48.

**Figure 6.**
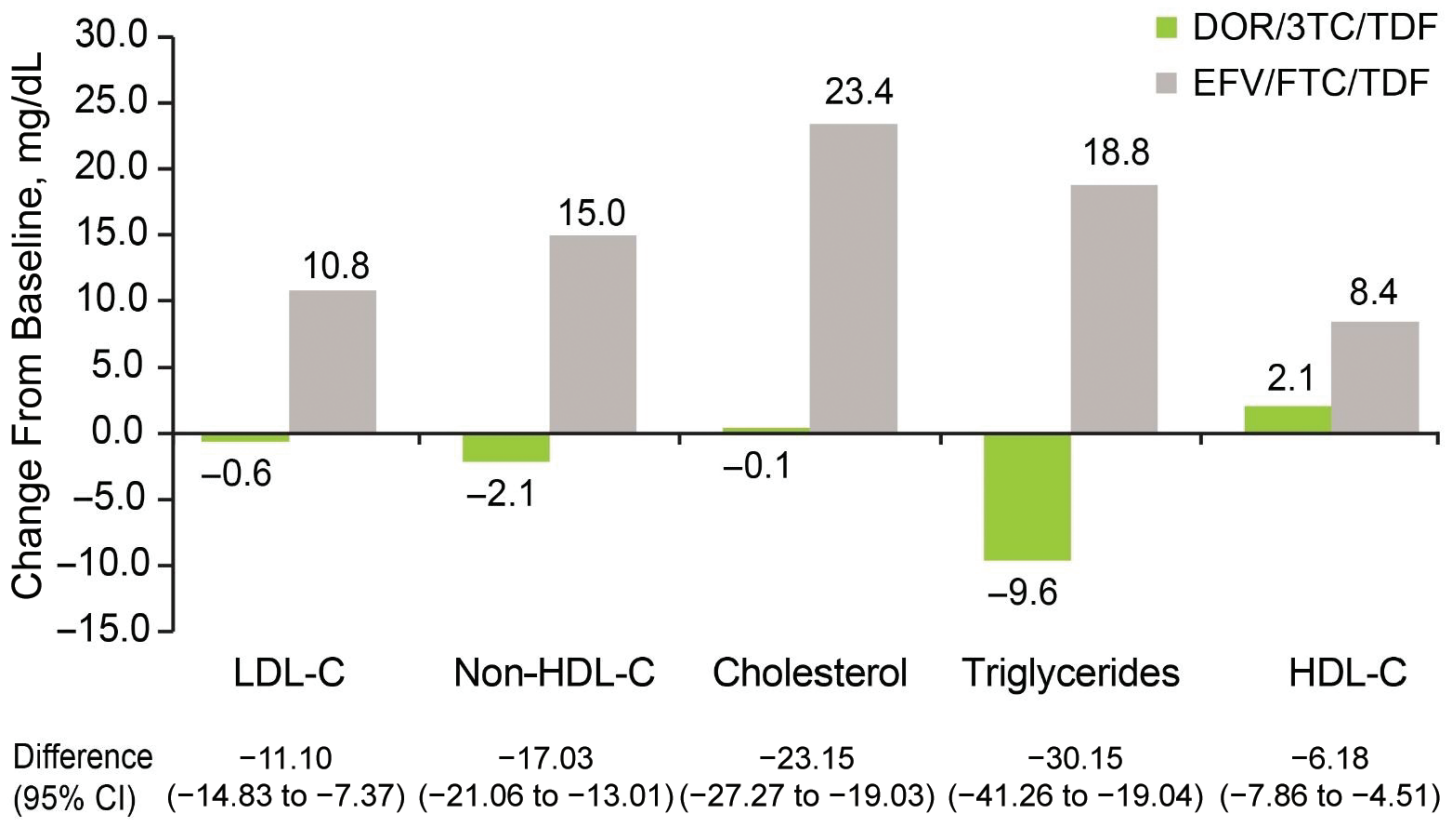
Mean change in fasting lipid levels at week 96. Abbreviations: 3TC, lamivudine; CI, confidence interval; DOR, doravirine; EFV, efavirenz; FTC, emtricitabine; HDL-C, high-density lipoprotein cholesterol; LDL-C, low-density lipoprotein cholesterol; non-HDL-C, non-HDL cholesterol; TDF, tenofovir disoproxil fumarate. (Between-group difference calculated as follows: DOR – EFV.)

Consistent with the week 48 findings, DOR/3TC/TDF had a favorable lipid profile with respect to mean changes from baseline in fasting low-density lipoprotein cholesterol and non-HDL-C levels at week 96 with minimal changes (−0.6 and −2.1 mg/dL, respectively) in the DOR/3TC/TDF group versus, compared with mean increases of 10.8 and 15.0 mg/dL, respectively, in the EFV/FTC/TDF group (Figure 6). A similar trend was observed for changes in baseline for fasting cholesterol, triglycerides, and HDL-C levels (Figure 6). The mean change from baseline in the total cholesterol/HDL-C ratio was however similar between arms: −0.12 for DOR/3TC/TDF and −0.10 for EFV/FTC/TDF (treatment difference, −0.04; 95% CI, −.23 to .15). In both the DOR/3TC/TDF and EFV/FTC/TDF groups, similar small mean increases in weight were noted from baseline to week 96 (2.1 kg and 1.6 kg, respectively).

## Discussion

The 96-week results of this phase 3 double-bind trial in treatment-naive adults living with HIV-1 support the noninferior efficacy of DOR/3TC/TDF relative to EFV/FTC/TDF, established at 48 weeks. At week 96, 77.5% of participants in the DOR/3TC/TDF group and 73.6% in the EFV/FTC/TDF group achieved HIV-1 RNA levels <50 copies/mL using the Food and Drug Administration snapshot approach, which is somewhat lower than the week 48 results for both groups (84.3% for DOR/3TC/TDF and 80.8% for EFV/FTC/TDF). One likely reason for the observed decrease in efficacy at week 96 is missing data. When missing data are excluded using the observed failure approach, higher virologic response rates were demonstrated for both treatment groups and were more closely aligned with what was observed at week 48 (83.7% for DOR/3TC/TDF and 85.9% for EFV/FTC/TDF). The virologic response rate in the EFV/FTC/TDF group at 96 weeks was consistent with rates reported for this regimen in previous clinical trials [12–14].

Antiviral response rates were consistent regardless of baseline demographic or prognostic factors. In both treatment groups, the virologic response rate was lower among participants with baseline HIV-1 RNA >100 000 copies/mL (71.0% for DOR/3TC/TDF and 79.7% for EFV/FTC/TDF) compared with those with baseline HIV-1 RNA ≤100 000 copies/mL (86.9% and 87.5%, respectively); this trend was also observed at week 48. Virologic response rates were also lower in patients with baseline CD4^+^ T-cell counts ≤200/μL (65% in the DOR/3TC/TDF vs 82% in EFV/FTC/TDF arm); however, only 12% of the total randomized population had low baseline CD4^+^ T-cell counts. In general, lower response rates in participants with high baseline viral load and low CD4^+^ T-cell counts have been observed in other clinical trials and across several antiretroviral classes [15, 16]. Immunologic responses, as measured by increases in CD4^+^ T-cell counts, were similar between the 2 treatment groups. Resistance to both DOR and EFV occurred primarily within the first 48 weeks of the trial. Between weeks 48 and 96, no new resistance to DOR and only 2 additional participants with resistance to EFV were observed. The DOR-associated NNRTI mutations identified in this trial were consistent with the mutations identified in previous in vitro resistance analyses [3, 4].

In this trial, the definition of PDVF and the criterion for required discontinuation were more stringent than in other recent trials. Other recent trials allowed participants to remain in the study despite meeting PDVF criteria or used a higher PDVF threshold of 200 or 400 copies/mL HIV-1 RNA [17–19]. In each treatment group, many participants with viral rebound had HIV-1 RNA ≤200 copies/mL at the viral failure confirmation visit, and, for the majority of those participants, HIV-1 RNA was between 50 and 100 copies/mL at that visit. However, even with this stringent definition for PDVF, rates of PDVF were low for both groups (9.3% for DOR/3TC/TDF and 7.7% for EFV/FTC/TDF).

A strength of our trial is the double-blind comparison between the 2 treatment groups through week 96. A potential limitation is that participants took 2 pills per day, 1 active drug and 1 placebo. with 1 pill taken while fasting, which may have been taken at different times, resulting in a twice-daily regimen. Although adherence to study therapy was high, it is possible that adherence would be improved with a single-pill, once-daily regimen. Furthermore, it is possible that this twice-daily dosing requirement of the study could have driven discontinuations in both arms.

The favorable safety profile of DOR/3TC/TDF was established in prior clinical studies [9, 10]. At week 96 of the DRIVE-AHEAD study, DOR/3TC/TDF continues to demonstrate a favorable safety and tolerability profile. Fewer participants in the DOR/3TC/TDF group experienced any AEs, drug-related AEs, or discontinuations due to AEs compared with the EFV/FTC/TDF group. Renal events were infrequent for both the DOR/3TC/TDF and EFV/FTC/TDF arms in this young cohort (median age, 31 years), with only 1 discontinuation due to a renal AE in the EFV/FTC/TDF group. The frequency of rash remained lower in the DOR/3TC/TDF group (5% vs 12% in the EFV/FTC/TDF group) and was consistent with rates previously reported for DOR [9]. Furthermore, no participants in the DOR/3TC/TDF group discontinued treatment due to rash. Week 96 data also continue to support the favorable lipid profile of DOR/3TC/TDF relative to EFV/FTC/TDF that was established at week 48. Because dyslipidemia occurs in a large proportion of people living with HIV and is a greater concern as patients age, it is important that antiretroviral medications do not exacerbate these conditions, especially for those already at high risk of cardiovascular disease.

At week 48, DOR/3TC/TDF exhibited favorable neuropsychiatric tolerability and was superior to EFV/FTC/TDF in the prespecified analysis of dizziness, sleep disorders/disturbances, and altered sensorium, showing fewer events in each of these categories. The week 96 results continue to show that DOR/3TC/TDF has a more favorable neuropsychiatric tolerability than EFV/FTC/TDF. EFV is known to be associated with neuropsychiatric AEs, and as a result it is possible that neuropsychiatric AEs were overreported in both arms in this blinded trial due to an ascertainment bias with EFV as comparator. In our trial the EFV/FTC/TDF arm still had a significantly higher percentage of neuropsychiatric AEs than the DOR/3TC/TDF arm (59% vs 26%, respectively).

In summary, the week 96 results of the DRIVE-AHEAD study show that the noninferior efficacy of DOR/3TC/TDF relative to EFV/FTC/TDF was maintained through week 96. Fewer patients developed treatment-emergent viral drug resistance to DOR than to EFV, with no additional resistance identified between weeks 48 and week 96 in the DOR group. In addition, DOR/3TC/TDF demonstrated a more favorable safety profile than EFV/FTC/TDF with fewer discontinuations due to AEs, fewer neuropsychiatric AEs, and a favorable lipid profile.

## Notes

***Author contributions.*** Conception, design or planning of the study: G. L., S. K., and E. M. Acquisition of data: C. O., K. E. S., J. M. M., O. S., G. L., S. K., C. H., and E. M. Analysis of data: G. L., G. J. H., C. H., E. M., and H. T. Interpretation of results: C. O., K. E. S., J. M. M., P. E. S., G. L., G. J. H., C. H., E. M., and H. T. Drafting of the manuscript: G. L. and C. H. Critical review or revision of the manuscript for important intellectual content: all authors.

***Acknowledgments***. The authors thank the participants and their families and caregivers for participating in this trial, along with all investigators and site personnel. Medical writing assistance was provided by Dean Campbell, PhD, and editorial assistance by Carol Zecca, BS, both from Merck & Co., Inc., Kenilworth, NJ, USA.

***Financial support.*** This work was supported by Merck Sharp & Dohme, a subsidiary of Merck & Co., Inc., Kenilworth, NJ, USA.

***Potential conflicts of interest.*** K. E. S., G. L., S. K., G. J. H., C. H., E. M., and H. T. are employees of Merck Sharp & Dohme, a subsidiary of Merck & Co., Inc., Kenilworth, NJ, USA, and may own stock and/or hold stock options in Merck. C. O. has received honoraria for participating in advisory boards and lecture fees from Merck, Gilead, ViiV, and Janssen, and her institution has received research grants from the same companies. J. M. M. has received honoraria for participating in advisory boards with Merck, Gilead, ViiV, BMS, and Teva, and his institution has received a research grant from Gilead. P. E. S. has received grants for scientific advisory boards with Gilead, Janssen, Merck, ViiV, and GSK. O. S. certifies no potential conflicts. All authors have submitted the ICMJE Form for Disclosure of Potential Conflicts of Interest. Conflicts that the editors consider relevant to the content of the manuscript have been disclosed.
